# Gut microbiota dysbiosis in post-cardiac arrest syndrome: mechanisms, clinical implications, and therapeutic perspectives

**DOI:** 10.3389/fmicb.2026.1893259

**Published:** 2026-08-07

**Authors:** Rui Liu, Junlong Wang

**Affiliations:** 1 Department of Emergency Medicine, The Second Affiliated Hospital of Zhejiang Chinese Medical University, Hangzhou, China; 2 Department of Urology, The First Affiliated Hospital of Zhejiang Chinese Medical University (Zhejiang Provincial Hospital of Chinese Medicine), Hangzhou, China

**Keywords:** dysbiosis, gut microbiota, gut–brain axis, intestinal barrier dysfunction, neuroinflammation, post-cardiac arrest syndrome

## Abstract

Post-cardiac arrest syndrome (PCAS) is a complex pathophysiological condition triggered by systemic ischemia–reperfusion injury following cardiac arrest and is frequently associated with multi-organ dysfunction and poor neurological outcomes. Emerging evidence suggests that the gut microbiota, increasingly recognized as a key regulator of host immunity, metabolism, and intestinal barrier integrity, may participate in the pathogenesis and progression of PCAS. Alterations in microbial composition and function following cardiac arrest have been associated with systemic inflammation, immune dysregulation, impaired intestinal barrier function, and gut–brain axis–mediated neurological injury. This narrative review summarizes current evidence regarding gut microbiota alterations after cardiac arrest and discusses their potential mechanistic links with PCAS progression. We further review emerging associations between microbial signatures and clinical outcomes and explore microbiota-targeted therapeutic approaches, including probiotics, prebiotics, and other microecological interventions. Given that direct evidence in PCAS remains limited, mechanistic insights derived from related critical illnesses are also discussed where biologically relevant. A better understanding of host–microbiota interactions may facilitate the identification of novel biomarkers and therapeutic targets and support the development of adjunctive strategies to improve outcomes after cardiac arrest.

## 1 Introduction

Post-cardiac arrest syndrome (PCAS) is a complex and multifaceted condition that develops after the restoration of spontaneous circulation following cardiac arrest. It comprises several interconnected pathological processes, including hypoxic–ischemic brain injury, myocardial dysfunction, systemic ischemia–reperfusion injury, and the persistent effects of the underlying cause of cardiac arrest. Despite advances in post-resuscitation care, patients with PCAS remain at high risk of severe neurological impairment, recurrent cardiovascular instability, multiple organ dysfunction, and death (Nolan et al., 2025; Hirsch et al., 2025; Sandroni et al., 2021; Hassager et al., 2026).

Among the organ systems affected by PCAS, the gastrointestinal tract is particularly vulnerable because of its sensitivity to hypoperfusion and oxygen deprivation. Intestinal ischemia–reperfusion injury may compromise mucosal barrier integrity, increase intestinal permeability, and facilitate the translocation of bacteria and microbial products into the systemic circulation. These gut-derived signals may amplify systemic inflammation and contribute to multiple organ dysfunction in critically ill patients (Deitch, 2012; Assimakopoulos et al., 2018; Potruch et al., 2022).

The gut microbiota is increasingly recognized as an important regulator of host metabolism, immune homeostasis, epithelial barrier function, and neuroimmune communication (Integrative Human Microbiome Project Research Network Consortium, 2019; Cryan et al., 2019; Tilg et al., 2020; Schluter et al., 2020). Alterations in microbial composition and function, collectively referred to as dysbiosis, have been implicated in systemic inflammation, immune dysregulation, and neurological injury through mechanisms involving microbial metabolites, intestinal barrier dysfunction, and the gut–brain axis (Cryan et al., 2019; Tilg et al., 2020; Schluter et al., 2020).

In PCAS, gut microbiota dysbiosis may represent both a consequence of global ischemia–reperfusion injury and a potential amplifier of post-resuscitation injury. However, direct clinical evidence regarding microbiota alterations in patients with PCAS remains limited. Consequently, many proposed mechanisms are inferred from experimental cardiac arrest models and studies of intestinal ischemia–reperfusion injury, sepsis, and general critical illness (Deitch, 2012; Assimakopoulos et al., 2018; Potruch et al., 2022; Otani et al., 2019).

This narrative review summarizes current evidence regarding gut microbiota alterations after cardiac arrest, examines the potential mechanisms linking dysbiosis with intestinal barrier dysfunction, systemic inflammation, immune dysregulation, and gut–brain axis-mediated neurological injury, and evaluates the potential clinical relevance of microbiota-related biomarkers and therapeutic strategies. Particular attention is given to the pathophysiological features that may distinguish PCAS-associated dysbiosis from the microbiota disturbances observed in other critical illnesses.

## 2 PCAS-relevant context of gut microbiota dysbiosis: distinctions from sepsis, causality, and temporal evolution

Although gut microbiota dysbiosis has been extensively investigated in sepsis and general critical illness, its potential role in PCAS should be interpreted within a distinct pathophysiological context. Sepsis-associated dysbiosis is shaped by infection, pathogen-associated inflammatory responses, antimicrobial exposure, nutritional interruption, and prolonged ICU-related ecological disruption. By contrast, PCAS begins with an abrupt cessation of systemic circulation followed by global ischemia–reperfusion injury after return of spontaneous circulation (ROSC). Microbiota alterations after cardiac arrest may therefore have a more abrupt onset and may initially be driven by intestinal hypoperfusion, mucosal ischemia–reperfusion injury, oxidative stress, mitochondrial dysfunction, endothelial injury, and early systemic inflammatory responses (Nolan et al., 2025; Deitch, 2012; Assimakopoulos et al., 2018; Potruch et al., 2022; Otani et al., 2019; Haak and Wiersinga, 2017; Peng et al., 2009).

### 2.1 Causality and bidirectional host–microbiota interactions

The causal relationship between gut microbiota dysbiosis and PCAS progression remains unresolved. Dysbiosis may initially develop as a consequence of systemic ischemia–reperfusion injury, hemodynamic instability, catecholamine and vasopressor exposure, antimicrobial therapy, delayed enteral nutrition, and other ICU interventions. Accordingly, microbiota alterations observed after ROSC cannot automatically be regarded as primary drivers of PCAS.

Nevertheless, dysbiosis may subsequently amplify post-resuscitation injury by aggravating intestinal barrier dysfunction, promoting the translocation of bacteria and microbial products, altering microbial metabolite production, and contributing to systemic inflammation, immune dysregulation, and gut–brain axis-mediated neuroinflammation. Pre-existing microbiota characteristics may also influence host resilience to ischemia–reperfusion injury and susceptibility to immune paralysis, secondary infection, and organ dysfunction (Deitch, 2012; Assimakopoulos et al., 2018; Potruch et al., 2022; Otani et al., 2019; McDonald et al., 2016; Lankelma et al., 2017; Haak and Wiersinga, 2017; Otani and Coopersmith, 2019; Peng et al., 2009).

These mechanisms are not mutually exclusive. Rather than being classified solely as either a cause or a consequence, gut microbiota dysbiosis may be more appropriately considered part of a dynamic and bidirectional host–microbiota injury loop after ROSC.

### 2.2 Temporal evolution after ROSC

The temporal evolution of microbiota alterations after ROSC may be clinically important. During the hyperacute phase, intestinal hypoperfusion and reperfusion injury may rapidly impair epithelial barrier integrity and alter luminal oxygen availability. These changes may favor facultative anaerobes and potentially pathogenic taxa while disrupting the intestinal environment required by obligate anaerobic commensals.

During the early post-resuscitation phase, systemic inflammation, oxidative stress, vasopressor exposure, antimicrobial therapy, and delayed enteral feeding may further reduce microbial diversity and deplete short-chain fatty acid (SCFA)-producing bacteria. During the later ICU phase, persistent dysbiosis, immune paralysis, nutritional disruption, and repeated antimicrobial exposure may increase susceptibility to secondary infection and multiple organ dysfunction (Hayakawa et al., 2011; Ojima et al., 2016; McDonald et al., 2016; Lankelma et al., 2017; Haak and Wiersinga, 2017; Otani and Coopersmith, 2019).

This proposed temporal framework remains largely based on evidence from ischemia–reperfusion models and general critical illness populations. Prospective studies with serial sampling after cardiac arrest are therefore required to determine the onset, magnitude, and persistence of microbiota alterations in PCAS. Microbiota-related biomarkers and interventions should also be evaluated according to the stage after ROSC rather than on the basis of isolated measurements.

### 2.3 Candidate PCAS-relevant microbial signatures

Microbial signatures unique to PCAS have not yet been definitively established. Candidate features may include an early reduction in α-diversity, expansion of Proteobacteria and Enterobacteriaceae, depletion of obligate anaerobes and SCFA-producing taxa, reduced butyrate-producing capacity, increased lipopolysaccharide-related inflammatory signaling, and alterations in microbial metabolites associated with neurological and cardiovascular injury (Ojima et al., 2016; McDonald et al., 2016; Lankelma et al., 2017; Haak and Wiersinga, 2017; Otani and Coopersmith, 2019; Haak et al., 2018a; Louis and Flint, 2017; Parada Venegas et al., 2019; Tan et al., 2014; Derrien et al., 2017; Cani and de Vos, 2017).

However, similar changes have also been observed in sepsis and other critical illnesses. These features should therefore be regarded as PCAS-relevant candidates rather than PCAS-specific biomarkers. Longitudinal studies comparing patients with PCAS against appropriately matched sepsis, cardiogenic shock, and non-infectious critical illness populations are needed to determine whether PCAS has a distinct microbial trajectory or predominantly shares a common critical illness-associated dysbiosis pattern.

## 3 Dynamic alterations of gut microbiota following PCAS

### 3.1 Acute loss of microbial diversity and compositional imbalance after ROSC

Available evidence indicates that cardiac arrest and subsequent ischemia–reperfusion injury may induce rapid alterations in gut microbiota composition, particularly during the early post-resuscitation phase. Experimental studies and observations from critically ill populations have demonstrated reductions in microbial α-diversity within the first several days after injury, as reflected by decreased Shannon and Chao1 indices, suggesting disruption of microbial ecosystem stability following systemic physiological stress (Hayakawa et al., 2011; Ojima et al., 2016; McDonald et al., 2016; Lankelma et al., 2017).

At the phylum level, several studies have reported reductions in beneficial commensal microbial communities accompanied by relative expansion of potentially pathogenic taxa, including members of the Proteobacteria phylum. Depletion of beneficial bacteria such as *Lactobacillus* and *Bifidobacterium* and increased abundance of opportunistic pathogens including *Enterobacteriaceae* have been observed in ischemia–reperfusion injury and critical illness models (Ojima et al., 2016; McDonald et al., 2016; Lankelma et al., 2017; Haak and Wiersinga, 2017). Although similar compositional alterations have been reported across different critical illnesses, the abrupt ischemic insult and subsequent reperfusion response characteristic of PCAS may contribute to distinct temporal dynamics of microbiota alterations.

**Figure 1:**
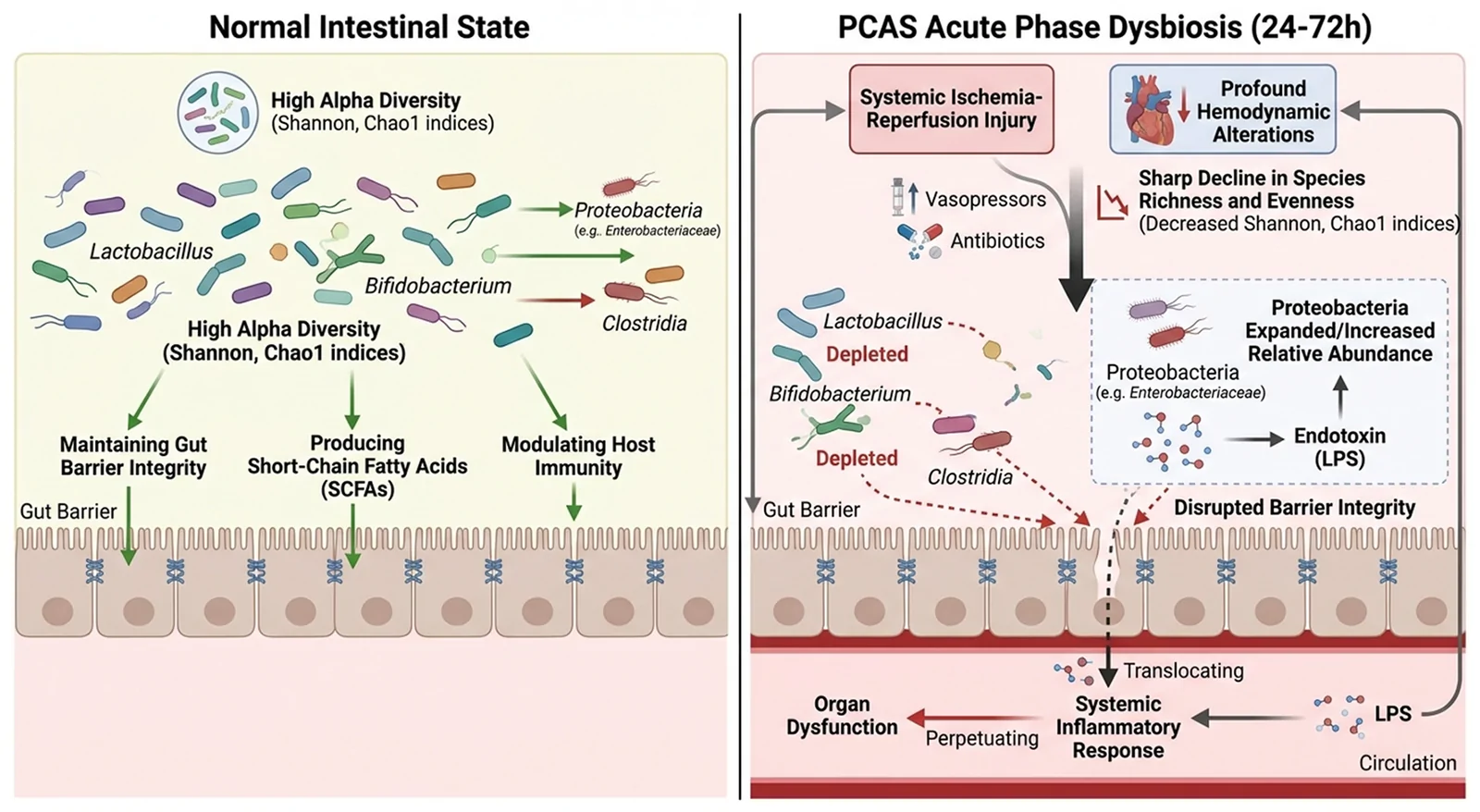
Acute-phase alterations in gut microbial diversity and microbial compositional imbalance following post-cardiac arrest syndrome. Following cardiac arrest and subsequent ischemia–reperfusion injury, gut microbial α-diversity may decrease during the early post-resuscitation period, indicating disruption of microbial ecosystem stability. Changes in microbial composition include depletion of beneficial commensal bacteria such as *Lactobacillus* and *Bifidobacterium*, accompanied by expansion of potentially pathogenic taxa including *Proteobacteria* and *Enterobacteriaceae*. These alterations may contribute to immune dysregulation, intestinal barrier dysfunction, and systemic inflammatory responses after cardiac arrest. This is an original schematic illustration created by the authors based on the literature cited in this section.

Altered microbial diversity and compositional imbalance have also been associated with markers of disease severity in critically ill patients, including organ dysfunction, inflammatory burden, and hemodynamic instability (McDonald et al., 2016; Lankelma et al., 2017; Haak and Wiersinga, 2017; Otani and Coopersmith, 2019). These findings suggest that gut microbiota profiles may potentially reflect the systemic physiological burden following cardiac arrest; however, direct validation in large PCAS-specific cohorts remains limited (Figure 1).

### 3.2 Depletion of functionally relevant microbial taxa after PCAS

Beyond broad taxonomic alterations, critical illness-associated dysbiosis may also involve selective depletion of microbial taxa that contribute to essential metabolic and barrier-supporting functions. Short-chain fatty acid (SCFA)-producing bacteria, including *Faecalibacterium, Roseburia*, and *Blautia*, have been reported to decrease in critically ill populations and experimental ischemia–reperfusion models (Haak et al., 2018a; Louis and Flint, 2017; Parada Venegas et al., 2019; Tan et al., 2014). Functional analyses further suggest reduced microbial pathways involved in SCFA biosynthesis and energy metabolism, potentially affecting host–microbiota interactions.

In parallel, bacterial taxa involved in mucin metabolism and epithelial support, particularly *Akkermansia muciniphila*, may also demonstrate reduced abundance under conditions of severe physiological stress (Derrien et al., 2017; Cani and de Vos, 2017). Given the role of these microorganisms in maintaining epithelial integrity and regulating immune homeostasis, depletion of such functionally relevant bacteria may contribute to impairment of host–microbe equilibrium.

**Figure 2:**
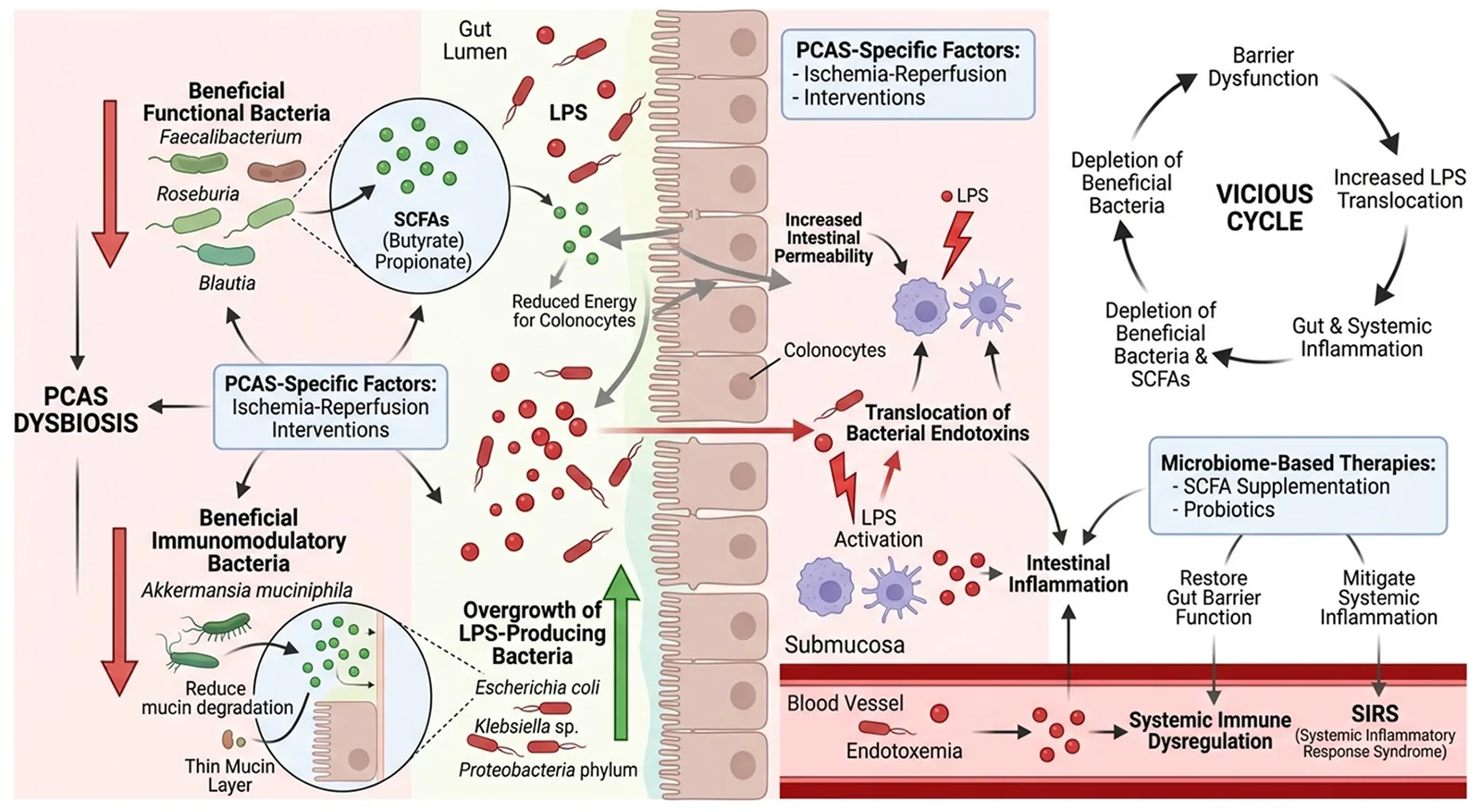
Functional depletion of metabolically and barrier-supporting microbial taxa following post-cardiac arrest syndrome. Gut microbiota alterations following cardiac arrest may involve depletion of short-chain fatty acid (SCFA)-producing bacteria, including *Faecalibacterium, Roseburia*, and *Blautia*, together with reduced abundance of *Akkermansia muciniphila*, a bacterium involved in mucin metabolism and epithelial support. Loss of these functionally relevant microbial taxa may contribute to impaired energy metabolism, intestinal barrier dysfunction, immune dysregulation, and altered host–microbiota homeostasis. This is an original schematic illustration created by the authors based on the literature cited in this section.

These observations suggest that microbiota alterations associated with PCAS may not solely reflect quantitative shifts in microbial abundance but may also involve loss of critical microbial functions. However, direct evidence from PCAS-specific cohorts remains limited, and further studies are required to clarify the functional significance of these alterations (Figure 2).

## 4 Mechanistic pathways linking gut microbiota dysbiosis to PCAS progression

### 4.1 Dysbiosis-associated systemic inflammation and immune dysregulation in PCAS

Gut microbiota dysbiosis may contribute to systemic inflammatory responses following cardiac arrest by promoting the translocation of microbe-associated molecular patterns (MAMPs), including lipopolysaccharide (LPS), into the circulation. Once translocated, LPS may activate Toll-like receptor 4 (TLR4) on innate immune cells and initiate downstream inflammatory signaling, thereby promoting proinflammatory cytokine production (Haak and Wiersinga, 2017; Akira et al., 2006; Takeuchi and Akira, 2010; Lu et al., 2008).

In parallel, depletion of short-chain fatty acid (SCFA)-producing bacteria may impair immune regulatory pathways. SCFAs, particularly butyrate, have been shown to support regulatory T-cell differentiation, maintain epithelial integrity, and modulate immune homeostasis (Parada Venegas et al., 2019; Tan et al., 2014; Furusawa et al., 2013). Reduced availability of SCFAs may therefore influence inflammatory balance and immune responses under conditions of severe physiological stress.

**Figure 3:**
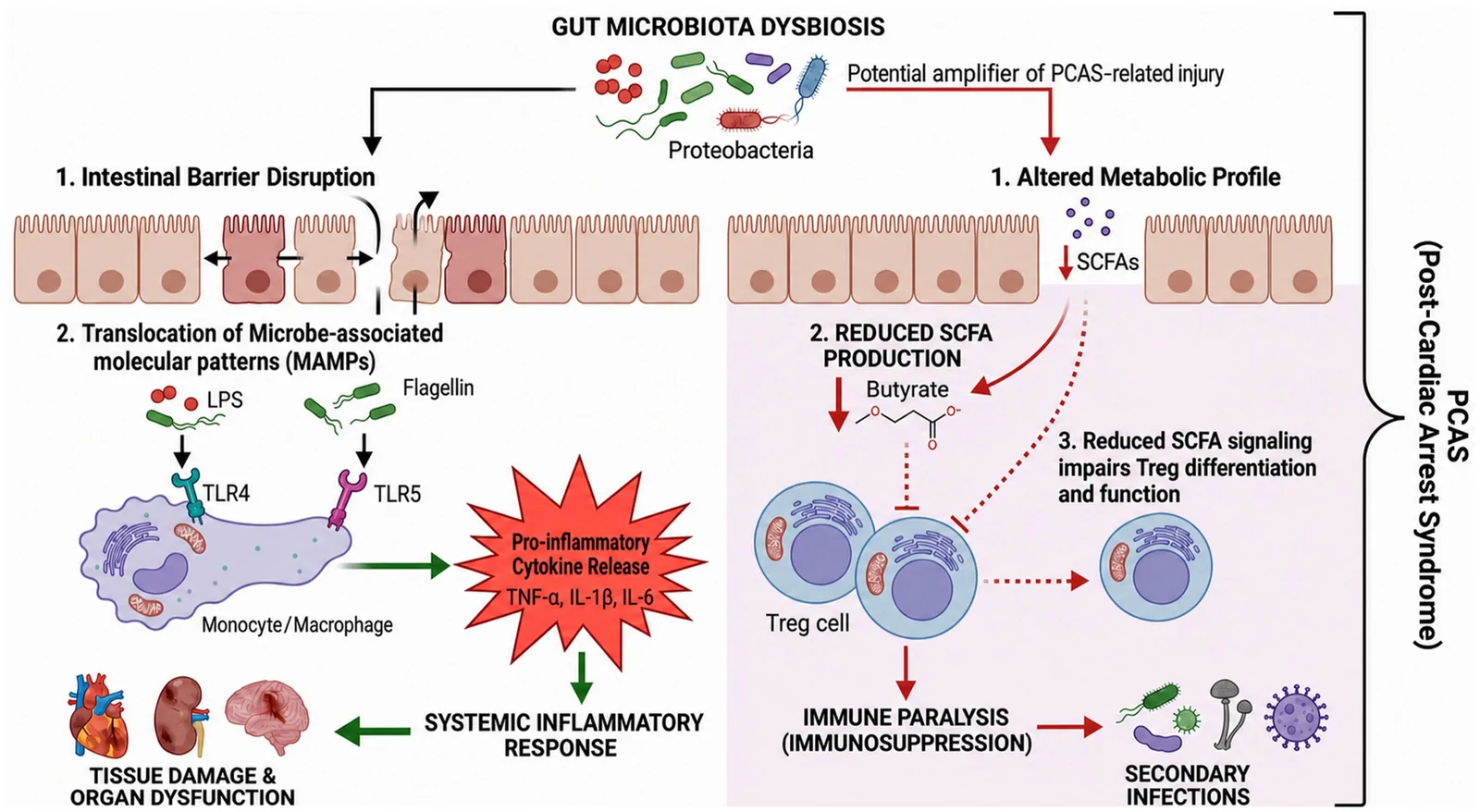
Proposed mechanisms linking gut microbiota dysbiosis with systemic inflammation and immune dysregulation in post-cardiac arrest syndrome. Gut microbiota dysbiosis following cardiac arrest may increase the translocation of microbe-associated molecular patterns (MAMPs), including lipopolysaccharide (LPS) and flagellin. Activation of the LPS–Toll-like receptor 4 (TLR4) and flagellin–Toll-like receptor 5 (TLR5) signaling pathways may promote pro-inflammatory cytokine release. Concurrent depletion of short-chain fatty acid-producing bacteria and reduced SCFA signaling may impair regulatory T-cell differentiation and function, potentially contributing to immune dysregulation, immune paralysis, and susceptibility to secondary infection during the post-resuscitation period. This is an original schematic illustration created by the authors based on the literature cited in this section.

PCAS is characterized by dynamic immune alterations involving both exaggerated inflammatory responses and subsequent immune suppression. Although gut microbiota dysbiosis has been proposed as a potential contributor to these changes, much of the mechanistic evidence currently originates from experimental studies, sepsis research, and cardiac arrest animal models rather than large clinical cohorts specifically involving PCAS patients (Delano and Ward, 2016; Hotchkiss et al., 2013; Adrie et al., 2002) (Figure 3).

### 4.2 Impaired intestinal barrier integrity and bacterial translocation after PCAS

Ischemia–reperfusion injury following cardiac arrest may directly damage intestinal epithelial cells, whereas gut microbiota dysbiosis may further aggravate intestinal barrier dysfunction. Short-chain fatty acid (SCFA) depletion has been associated with impaired colonocyte energy metabolism and reduced expression of tight junction proteins, including occludin and claudins, thereby compromising epithelial integrity (Louis and Flint, 2017; Parada Venegas et al., 2019; Peng et al., 2009; Kelly et al., 2015).

Disruption of intestinal barrier function may facilitate translocation of luminal bacteria and microbial products across the intestinal epithelium into mesenteric lymph nodes and systemic circulation. These gut-derived factors may subsequently act as endogenous sources of inflammatory stimuli and contribute to systemic inflammatory responses (Deitch, 2012; Assimakopoulos et al., 2018; Potruch et al., 2022).

**Figure 4:**
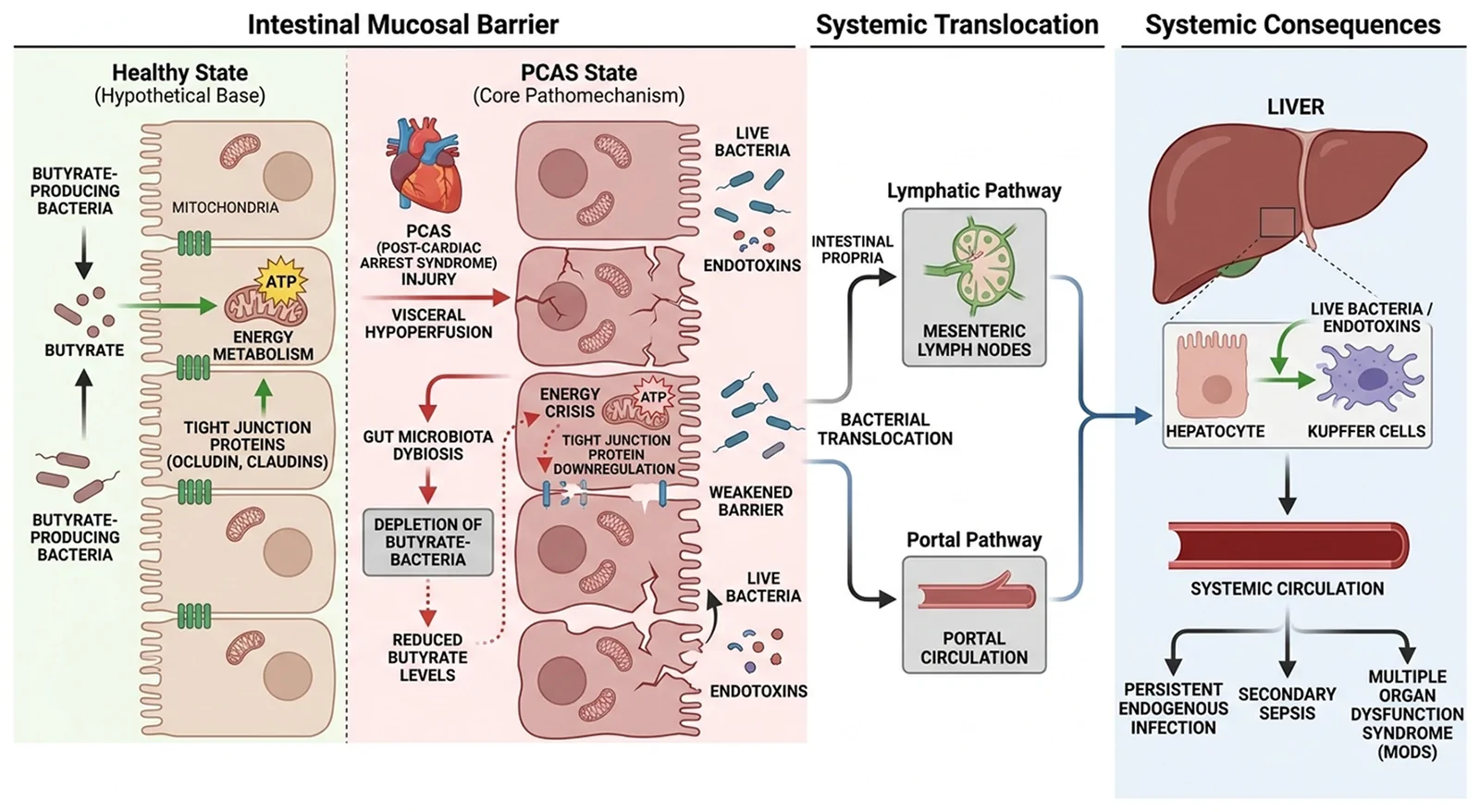
Proposed mechanisms linking intestinal barrier dysfunction and bacterial translocation following post-cardiac arrest syndrome. Cardiac arrest-induced ischemia–reperfusion injury together with gut microbiota dysbiosis may impair intestinal epithelial integrity through reduced short-chain fatty acid (SCFA) availability and altered tight junction protein expression. Disruption of epithelial barrier function may facilitate translocation of luminal bacteria and microbial products into mesenteric lymph nodes and systemic circulation, potentially contributing to systemic inflammation and multiple organ dysfunction during the post-resuscitation period. This is an original schematic illustration created by the authors based on the literature cited in this section.

Accumulating evidence from critical illness and experimental ischemia–reperfusion models suggests that bacterial translocation may contribute to secondary infection, immune dysregulation, and multiple organ dysfunction. However, direct evidence regarding these mechanisms specifically in PCAS remains limited (Deitch, 2012; Otani et al., 2019; Haak and Wiersinga, 2017) (Figure 4).

### 4.3 Gut–brain axis involvement in neurological injury and recovery after PCAS

Neurological injury remains the principal determinant of long-term prognosis in patients with PCAS, rendering gut–brain axis interactions particularly relevant in this setting. Gut microbiota dysbiosis may contribute to systemic inflammatory responses through increased production and translocation of pro-inflammatory mediators. Elevated circulating cytokines, including interleukin-1β (IL-1β), interleukin-6 (IL-6), and tumor necrosis factor-α (TNF-α), may subsequently influence central nervous system signaling pathways and contribute to neuroinflammation (Cryan et al., 2019; Fung et al., 2017; Erny et al., 2017; Sampson and Mazmanian, 2015).

In addition, microbiota-derived metabolites, particularly short-chain fatty acids (SCFAs), have been implicated in modulation of microglial activation, oxidative stress responses, and neuronal survival. Reduced production of neuroprotective metabolites under dysbiotic conditions may therefore influence neurological recovery following systemic injury (Parada Venegas et al., 2019; Dalile et al., 2019; Silva et al., 2020).

**Figure 5:**
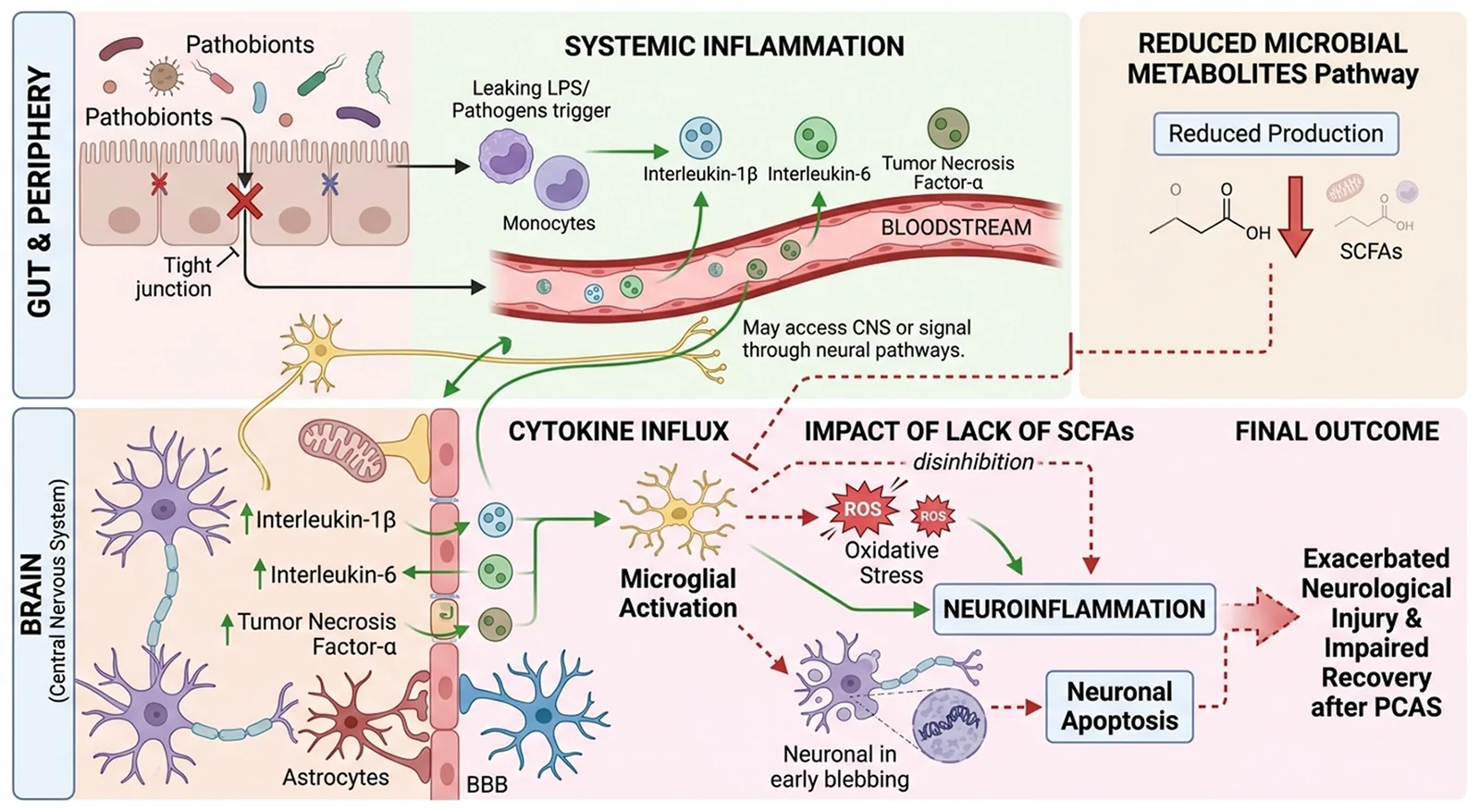
Proposed interactions between gut microbiota dysbiosis and neurological injury through the gut–brain axis following post-cardiac arrest syndrome. Gut microbiota dysbiosis following cardiac arrest may contribute to systemic inflammatory responses and altered production of microbiota-derived metabolites. Elevated circulating inflammatory mediators and reduced short-chain fatty acid (SCFA) availability may influence central nervous system signaling pathways, microglial activation, oxidative stress, and neuronal survival. These interactions may collectively contribute to neuroinflammation and neurological injury during the post-resuscitation period. This is an original schematic illustration created by the authors based on the literature cited in this section.

Experimental studies suggest that modulation of gut microbiota composition may attenuate neuroinflammatory responses and improve neurological outcomes in models of neurological injury. However, direct evidence specifically linking gut microbiota alterations with neurological recovery after PCAS remains limited and requires further investigation (Benakis et al., 2016; Singh et al., 2016) (Figure 5).

## 5 Candidate microbiota-related biomarkers and clinical implications in PCAS

### 5.1 Candidate microbial signatures and their potential prognostic relevance

Emerging evidence suggests that gut microbiota-related biomarkers may have potential value for disease stratification and prognostic assessment in critically ill patients, although direct evidence in PCAS populations remains limited. At present, the most biologically plausible and clinically promising microbial indicators include reduced microbial diversity, expansion of potentially pathogenic taxa, depletion of beneficial commensal bacteria and SCFA-producing taxa, and alterations in microbiota-derived metabolites and functional pathways.

Among these candidates, reduced α-diversity, reflected by lower Shannon and Chao1 indices, represents one of the most consistently reported microbiota alterations in critical illness and severe systemic physiological stress. Although it is not specific to PCAS, decreased microbial diversity may reflect loss of microbial ecosystem stability and has been associated with greater disease severity, organ dysfunction, inflammatory burden, and hemodynamic instability in critically ill populations (McDonald et al., 2016; Lankelma et al., 2017). Therefore, microbial diversity indices may serve as broad indicators of host–microbiota disruption during the post-resuscitation period.

Expansion of potentially pathogenic taxa, particularly Proteobacteria and Enterobacteriaceae, may also have clinical relevance because these microorganisms are closely related to endotoxin production, impaired colonization resistance, and increased risk of infectious complications (McDonald et al., 2016; Lankelma et al., 2017; Haak and Wiersinga, 2017; Dickson, 2016; Dickson et al., 2016). In contrast, depletion of beneficial commensal bacteria, including *Lactobacillus* and *Bifidobacterium*, may reflect impaired microbial homeostasis and reduced host defense capacity. These taxonomic changes may be especially relevant in PCAS patients who are vulnerable to secondary infections, immune dysregulation, and multiple organ dysfunction.

Functional microbial biomarkers may provide additional clinical information beyond taxonomic composition. Depletion of SCFA-producing bacteria, including *Faecalibacterium, Roseburia*, and *Blautia*, together with reduced butyrate-related metabolic capacity, may indicate impaired epithelial energy metabolism, weakened intestinal barrier integrity, and altered immune regulation (Haak et al., 2018a; Louis and Flint, 2017; Parada Venegas et al., 2019; Tan et al., 2014; Furusawa et al., 2013; Peng et al., 2009; Kelly et al., 2015). Because intestinal barrier dysfunction and systemic inflammation are central components of PCAS pathophysiology, SCFA-related microbial signatures may have particular translational potential.

Microbial products and metabolites may also serve as candidate biomarkers. Reduced butyrate availability, potentially increased circulating LPS or LPS-related inflammatory signaling, and altered trimethylamine N-oxide (TMAO) levels may reflect interactions among dysbiosis, systemic inflammation, gut–brain axis dysfunction, and cardiovascular complications (Cryan et al., 2019; Haak and Wiersinga, 2017; Parada Venegas et al., 2019; Akira et al., 2006; Takeuchi and Akira, 2010; Lu et al., 2008; Fung et al., 2017; Erny et al., 2017; Sampson and Mazmanian, 2015; Dalile et al., 2019; Silva et al., 2020; Tang et al., 2013; Brown and Hazen, 2015; Janeiro et al., 2018). However, these metabolite-based markers remain exploratory in the context of PCAS and require prospective validation.

Overall, microbiota-based biomarkers with the greatest current clinical potential include α-diversity indices, Proteobacteria/Enterobacteriaceae expansion, depletion of SCFA-producing bacteria, and SCFA/LPS-related functional signatures. Nevertheless, most available evidence is extrapolated from sepsis, ischemia–reperfusion injury, and general critical illness studies. Large longitudinal PCAS-specific cohorts integrating metagenomics, metabolomics, inflammatory markers, neurological outcomes, and survival endpoints are needed before these biomarkers can be translated into routine clinical practice.

### 5.2 Potential relationships between gut microbiota and common PCAS complications

Gut microbiota dysbiosis may be associated with several complications commonly observed following PCAS. Secondary infections, including ventilator-associated pneumonia, catheter-related bloodstream infections, and other nosocomial infections, frequently occur in critically ill patients after cardiac arrest. Disruption of gut microbial homeostasis together with reduced colonization resistance against opportunistic pathogens may facilitate microbial translocation and systemic dissemination of potentially pathogenic microorganisms, thereby increasing susceptibility to infection (Lankelma et al., 2017; Haak and Wiersinga, 2017; Dickson, 2016; Dickson et al., 2016).

Regarding cardiovascular complications, increasing attention has been directed toward the concept of the gut–heart axis. Gut microbiota-derived metabolites and translocated microbial products may contribute to systemic inflammatory responses that influence myocardial function and vascular homeostasis. In particular, trimethylamine-N-oxide (TMAO), a microbiota-derived metabolite, has been associated with cardiovascular dysfunction and adverse cardiovascular outcomes in multiple disease settings (Tang et al., 2013; Brown and Hazen, 2015; Janeiro et al., 2018). However, direct evidence linking TMAO with myocardial dysfunction specifically following PCAS remains limited.

Acute kidney injury (AKI), another common complication after cardiac arrest, may also be influenced by interactions between gut microbiota dysbiosis, systemic inflammation, and intestinal barrier dysfunction. Experimental studies suggest that gut-derived inflammatory mediators and microbial products may participate in pathways involved in renal injury and progression of organ dysfunction (Yang et al., 2018; Ramezani and Raj, 2014; Meijers and Evenepoel, 2011).

Collectively, these findings suggest that gut microbiota alterations may influence multiple complications observed following PCAS through interconnected inflammatory and metabolic pathways. However, the underlying mechanisms and their clinical significance in PCAS require further validation in prospective studies. The major gut microbiota alterations and their potential pathophysiological implications in PCAS are summarized in Table 1.

**Table 1:** Major gut microbiota alterations and proposed pathophysiological implications in post-cardiac arrest syndrome.

Gut microbiota alteration	Representative taxa/metabolites	Proposed biological effects	Potential clinical implications in PCAS	Supporting references
Reduced microbial diversity	↓ Shannon index; ↓ Chao1 index	Loss of microbial ecosystem stability	Associated with severe physiological stress and organ dysfunction	Hayakawa et al., 2011; Ojima et al., 2016; McDonald et al., 2016; Lankelma et al., 2017
Expansion of potentially pathogenic microorganisms	↑ Proteobacteria; ↑ Enterobacteriaceae	Increased exposure to MAMPs, including LPS, and enhanced pattern-recognition receptor signaling	May contribute to systemic inflammation and infection susceptibility	Ojima et al., 2016; McDonald et al., 2016; Lankelma et al., 2017; Haak and Wiersinga, 2017; Akira et al., 2006; Takeuchi and Akira, 2010; Lu et al., 2008; Dickson, 2016; Dickson et al., 2016
Depletion of beneficial commensal bacteria	↓*Lactobacillus*; ↓*Bifidobacterium*	Reduced colonization resistance and immune modulation	May facilitate dysbiosis progression and impaired host defense	Ojima et al., 2016; McDonald et al., 2016; Lankelma et al., 2017; Haak and Wiersinga, 2017; Dickson, 2016; Dickson et al., 2016
Reduced SCFA-producing bacteria	↓*Faecalibacterium*; ↓*Roseburia*; ↓*Blautia*	Reduced SCFA production and impaired epithelial energy metabolism	May contribute to intestinal barrier dysfunction and immune dysregulation	Haak et al., 2018a; Louis and Flint, 2017; Parada Venegas et al., 2019; Tan et al., 2014; Furusawa et al., 2013; Peng et al., 2009; Kelly et al., 2015
Reduced mucin-associated bacteria	↓ Akkermansia muciniphila	Impaired mucosal integrity and epithelial support	May increase intestinal permeability	Derrien et al., 2017; Cani and de Vos, 2017
Altered microbial products and metabolites	↓ Butyrate; potentially increased LPS-related signaling; altered TMAO levels	Enhanced LPS–TLR4 signaling and altered metabolic and immune responses	May influence neurological injury and cardiovascular dysfunction	Cryan et al., 2019; Haak and Wiersinga, 2017; Parada Venegas et al., 2019; Akira et al., 2006; Takeuchi and Akira, 2010; Lu et al., 2008; Fung et al., 2017; Erny et al., 2017; Sampson and Mazmanian, 2015; Dalile et al., 2019; Silva et al., 2020; Tang et al., 2013; Brown and Hazen, 2015; Janeiro et al., 2018
Intestinal barrier disruption	Tight junction impairment, occludin/claudin changes	Increased bacterial translocation	May contribute to systemic inflammation and multiple organ dysfunction	Deitch, 2012; Assimakopoulos et al., 2018; Potruch et al., 2022; Otani et al., 2019; Haak and Wiersinga, 2017; Louis and Flint, 2017; Parada Venegas et al., 2019; Peng et al., 2009; Kelly et al., 2015
Gut–brain axis dysregulation	Cytokines; microbial metabolites	Microglial activation and neuroinflammation	May influence neurological recovery and long-term prognosis	Cryan et al., 2019; Parada Venegas et al., 2019; Fung et al., 2017; Erny et al., 2017; Sampson and Mazmanian, 2015; Dalile et al., 2019; Silva et al., 2020; Benakis et al., 2016; Singh et al., 2016

Alterations in gut microbiota following cardiac arrest may involve reduced microbial diversity, depletion of beneficial microbial taxa, altered microbial metabolites, and intestinal barrier dysfunction. These microbiota-related changes may contribute to inflammatory responses, immune dysregulation, and organ injury after cardiac arrest.

LPS, lipopolysaccharide; MAMP, microbe-associated molecular pattern; PCAS, post-cardiac arrest syndrome; SCFA, short-chain fatty acid; TLR4, Toll-like receptor 4; TMAO, trimethylamine N-oxide.

## 6 Targeting gut microbiota in PCAS: therapeutic strategies and prospects

### 6.1 Potential applications of microecological regulation therapy

Microecological regulation therapies, including probiotics, prebiotics, synbiotics, selective digestive decontamination (SDD), and fecal microbiota transplantation (FMT), have emerged as potential approaches for modulating gut microbiota disturbances in critically ill populations and may represent promising strategies for PCAS management (Otani et al., 2019; Haak et al., 2018b; Dickson, 2016; Valdes et al., 2018).

Probiotics and prebiotics may contribute to restoration of microbial homeostasis through enhancement of beneficial microbial communities and increased production of microbiota-derived metabolites such as short-chain fatty acids (SCFAs). Experimental studies and observations from critical illness models suggest that these interventions may reduce inflammatory responses, preserve intestinal barrier integrity, and influence immune regulation (Parada Venegas et al., 2019; Sanders et al., 2019; Kristensen et al., 2016; Wilkins and Sequoia, 2017). However, direct evidence regarding their efficacy specifically in PCAS remains limited.

SDD, involving administration of non-absorbable antimicrobial agents to reduce colonization by potentially pathogenic microorganisms, has demonstrated benefits in reducing infection rates in selected intensive care unit populations (Meessen et al., 2009; Daneman et al., 2013). Nevertheless, its use in PCAS requires careful consideration because of concerns regarding disruption of microbial ecology and emergence of antimicrobial resistance.

FMT represents a more comprehensive approach for restoring microbial composition and function and has demonstrated clinical efficacy in recurrent *Clostridioides difficile* infection (van Nood et al., 2013). Increasing interest has also emerged regarding potential applications of FMT in inflammatory and critical illness conditions (Ooijevaar et al., 2019; Allegretti et al., 2019). However, in the setting of PCAS, FMT remains largely experimental, and important concerns regarding safety, infection transmission, hemodynamic instability, and patient selection remain unresolved.

Collectively, microecological regulation therapies may represent promising adjunctive strategies for modulating gut microbiota disturbances after cardiac arrest. However, their clinical applicability in PCAS requires further investigation through mechanistic studies and well-designed prospective clinical trials.

### 6.2 Emerging and precision microbiome-targeted strategies

Beyond conventional probiotics, prebiotics, synbiotics, SDD, and FMT, several emerging microbiome-targeted strategies may have potential relevance for PCAS, including postbiotics, microbial metabolites, engineered probiotics, bacteriophage therapy, and personalized microbiome-based interventions (Sanders et al., 2019; van Nood et al., 2013; Ooijevaar et al., 2019; Allegretti et al., 2019; Cani, 2018; Gulliver et al., 2022; Hsu et al., 2019). Postbiotics—preparations of inanimate microorganisms and/or their components that confer a health benefit—may provide immunomodulatory and barrier-protective effects without the risks associated with administering live microorganisms to critically ill patients (Cani, 2018; Gulliver et al., 2022). This feature may be particularly relevant in PCAS patients with hemodynamic instability, immune dysregulation, impaired intestinal barrier integrity, and high risk of secondary infection.

Microbial metabolites and metabolite-based therapies represent another promising direction. Among these, SCFAs, particularly butyrate, may support colonocyte energy metabolism, strengthen tight junction integrity, regulate inflammatory responses, and influence gut–brain axis signaling (Louis and Flint, 2017; Parada Venegas et al., 2019; Tan et al., 2014; Furusawa et al., 2013; Peng et al., 2009; Kelly et al., 2015; Dalile et al., 2019; Silva et al., 2020). Therefore, SCFA supplementation or strategies designed to restore SCFA-producing microbial communities may theoretically attenuate intestinal barrier dysfunction and systemic inflammation after ROSC. However, the optimal formulation, route of administration, timing, dose, and safety profile of SCFA-based therapy in PCAS remain unknown.

Engineered probiotics may provide more targeted therapeutic functions than conventional probiotic strains, such as local delivery of anti-inflammatory molecules, enhancement of epithelial barrier repair, or modulation of oxidative stress and immune responses (Sanders et al., 2019; Cani, 2018; Gulliver et al., 2022). Nevertheless, their application in PCAS remains highly experimental and raises important concerns regarding biosafety, genetic stability, uncontrolled colonization, horizontal gene transfer, and regulatory approval.

Bacteriophage therapy may offer a precision approach for selectively reducing pathogenic or opportunistic bacterial overgrowth while preserving beneficial commensal organisms (Hsu et al., 2019). This strategy may be relevant in ICU patients with expansion of Enterobacteriaceae or other potentially pathogenic taxa. However, phage therapy requires pathogen identification, individualized phage selection, careful monitoring of bacterial resistance, and evaluation of immune responses to phage administration (Hsu et al., 2019).

Personalized microbiome therapy may represent a future direction for PCAS management. Because gut microbiota composition varies substantially among individuals and changes dynamically during critical illness, a universal intervention is unlikely to be effective for all patients (McDonald et al., 2016; Lankelma et al., 2017; Zmora et al., 2018). Integration of metagenomics, metabolomics, clinical phenotyping, antibiotic exposure, nutritional status, inflammatory markers, and neurological outcomes may help identify patients who are most likely to benefit from microbiota-targeted interventions (Lloyd-Price et al., 2016; Gilbert et al., 2018; Knight et al., 2018; Zmora et al., 2018; Cani, 2018; Gulliver et al., 2022).

Safety is a central consideration when translating microbiome-based therapies to PCAS. Critically ill patients may have impaired intestinal barrier function, immune paralysis, central venous catheters, broad-spectrum antibiotic exposure, and hemodynamic instability, all of which may increase the risk of bloodstream infection, translocation of administered organisms, antimicrobial resistance, and unpredictable host–microbe interactions (Otani et al., 2019; Haak et al., 2018b; Dickson, 2016; Meessen et al., 2009; Daneman et al., 2013; van Nood et al., 2013; Ooijevaar et al., 2019; Allegretti et al., 2019). Therefore, live biotherapeutic products, FMT, and engineered microbial therapies should be evaluated cautiously in controlled clinical trials before routine clinical use.

The timing of intervention after ROSC may also determine therapeutic efficacy and safety. In the hyperacute phase, priority should be given to hemodynamic stabilization, optimization of perfusion, and avoidance of additional intestinal injury (Nolan et al., 2025; Adrie et al., 2002; Reintam Blaser et al., 2012). In the early post-resuscitation phase, strategies that preserve barrier integrity, minimize unnecessary antibiotic exposure, and support enteral nutrition may be more feasible (Nolan et al., 2025; Adrie et al., 2002; Haak et al., 2018b; Reintam Blaser et al., 2012; McClave et al., 2016; Wischmeyer, 2017; Singer et al., 2023). In the later ICU phase, microbiota-restorative strategies, including targeted prebiotics, postbiotics, metabolite supplementation, or individualized microbial therapies, may be considered for patients with persistent dysbiosis, secondary infection risk, or prolonged organ dysfunction (Otani et al., 2019; Parada Venegas et al., 2019; Haak et al., 2018b; Dickson, 2016; Valdes et al., 2018; Sanders et al., 2019; McClave et al., 2016; Wischmeyer, 2017; Singer et al., 2023; Demehri et al., 2015; Cani, 2018; Gulliver et al., 2022; Hsu et al., 2019). This stage-specific approach may help align microbiota-based interventions with the evolving pathophysiology of PCAS.

### 6.3 Protective effects of comprehensive supportive therapies on gut microbiota

Comprehensive supportive care following cardiac arrest may influence gut microbiota homeostasis and potentially mitigate dysbiosis-associated complications. Optimization of hemodynamic status and tissue perfusion during post-resuscitation care may help maintain intestinal blood flow and reduce intestinal ischemic injury. Excessive vasoconstrictive support may adversely affect splanchnic perfusion and compromise intestinal barrier integrity, highlighting the importance of balanced circulatory management (Nolan et al., 2025; Adrie et al., 2002; Reintam Blaser et al., 2012).

Nutritional support may also influence gut microbiota composition and intestinal homeostasis. Early enteral nutrition, particularly when clinically feasible, may preserve intestinal mucosal integrity and provide substrates for commensal microbiota, thereby supporting microbial metabolic activity and epithelial function (McClave et al., 2016; Wischmeyer, 2017; Singer et al., 2023). In contrast, prolonged reliance on parenteral nutrition has been associated with alterations in gut microbial communities and impaired barrier function in some settings (Demehri et al., 2015).

Antibiotic exposure represents another major factor influencing microbiota composition in critically ill patients. Broad-spectrum antimicrobial therapy may substantially alter gut microbial ecology and reduce colonization resistance against opportunistic pathogens (Dickson, 2016). Appropriate antimicrobial stewardship, including strict indication assessment and timely de-escalation based on microbiological findings, may minimize unintended disruption of beneficial microbial communities (Haak et al., 2018b).

Collectively, these integrated supportive approaches may contribute to maintenance of gut microbial homeostasis and modulation of host inflammatory responses during the post-resuscitation period. However, direct evidence regarding their microbiota-related effects specifically in PCAS remains limited.

### 6.4 Future research directions and challenges

Further investigation is required to improve understanding of the role of gut microbiota in PCAS and to facilitate translation of microbiota-related findings into clinical practice. Future mechanistic studies employing germ-free and genetically modified animal models, together with integrated multi-omics approaches including metagenomics, metabolomics, transcriptomics, and proteomics, may help clarify interactions between gut microbial alterations and host responses following cardiac arrest (Lloyd-Price et al., 2016; Gilbert et al., 2018; Knight et al., 2018).

Large multicenter longitudinal clinical studies are also needed to characterize temporal patterns of microbiota alterations after cardiac arrest and to identify microbial or metabolite-based biomarkers potentially associated with disease severity and clinical outcomes (Johnson et al., 2019; Zmora et al., 2018). Such studies may provide insights into patient stratification and improve understanding of host–microbiota interactions during different phases of PCAS.

Emerging microbiota-targeted approaches, including tailored probiotic formulations, prebiotic interventions, bacteriophage-based therapies, and other precision microbiome strategies, may represent potential future therapeutic directions (Cani, 2018; Gulliver et al., 2022; Hsu et al., 2019). However, multiple challenges remain, including substantial interindividual heterogeneity, dynamic microbiota alterations during critical illness, safety concerns, and incomplete understanding of host–microbe interactions.

Optimization of treatment timing, administration strategies, and patient selection may also influence future clinical applications. Collaborative efforts integrating clinical medicine, microbiology, bioengineering, and computational approaches will likely be necessary to advance this field and support development of microbiota-based interventions in PCAS.

**Figure 6:**
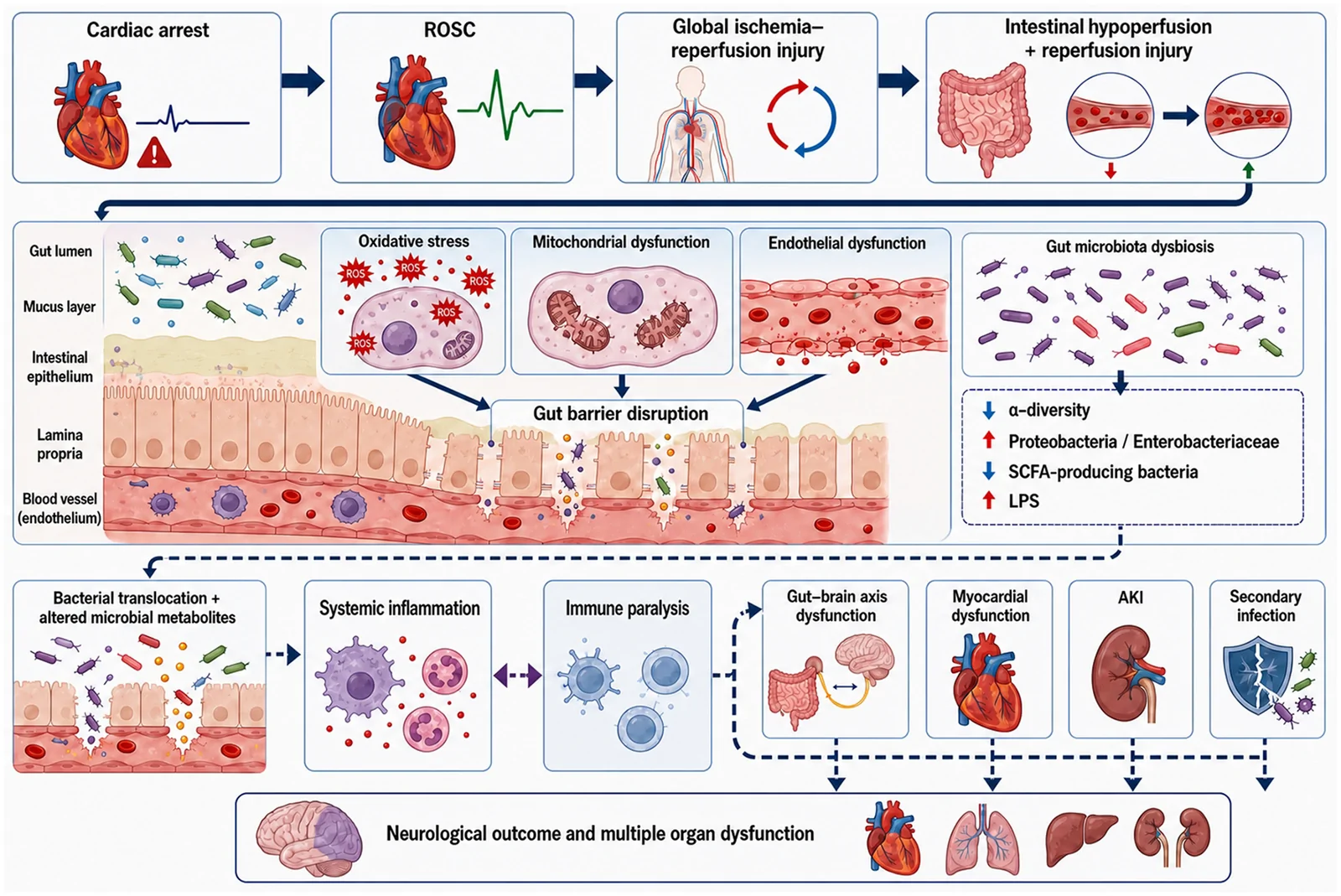
PCAS-specific integrated framework linking ROSC, intestinal hypoperfusion, gut microbiota dysbiosis, and downstream organ dysfunction. Cardiac arrest followed by return of spontaneous circulation (ROSC) initiates global ischemia–reperfusion injury. Intestinal hypoperfusion and reperfusion injury may induce oxidative stress, mitochondrial dysfunction, endothelial dysfunction, and epithelial barrier damage. These changes may promote gut microbiota dysbiosis, characterized by reduced microbial diversity, expansion of potentially pathogenic taxa, depletion of SCFA-producing bacteria, increased LPS-related signaling, and altered microbial metabolites. Dysbiosis and barrier disruption may further contribute to bacterial translocation, systemic inflammation, immune paralysis, gut–brain axis dysfunction, myocardial dysfunction, acute kidney injury, secondary infection, and multiple organ dysfunction. Solid arrows indicate mechanisms supported by evidence from PCAS, ischemia–reperfusion injury, sepsis, or critical illness studies; dashed arrows indicate hypothetical or insufficiently validated pathways in PCAS. This is an original schematic illustration created by the authors based on the literature cited in Sections 1–5.

The proposed interactions linking cardiac arrest-induced systemic injury, gut microbiota alterations, and downstream organ dysfunction are summarized in Figure 6.

## 7 Conclusion

Emerging evidence indicates that gut microbiota alterations may represent an important component of the pathophysiological processes occurring after cardiac arrest. Compared with infection-driven critical illnesses, PCAS is characterized by abrupt global ischemia–reperfusion injury and dynamic systemic responses during the early post-resuscitation period, which may influence gut microbial composition and function. Alterations in microbial diversity and abundance have been associated with inflammatory responses, immune dysregulation, and intestinal barrier dysfunction observed following cardiac arrest.

Interactions between gut microbiota and host responses may also have implications for neurological injury and recovery, which remain major determinants of long-term prognosis in PCAS. Through mechanisms involving systemic inflammation, microbial metabolites, and gut–brain axis signaling pathways, gut microbiota dysbiosis may contribute to neuroinflammatory responses following cardiac arrest.

However, current mechanistic understanding remains largely derived from experimental studies and evidence extrapolated from sepsis and general critical illness populations. Direct evidence specifically involving PCAS patients remains limited, and microbial signatures associated with disease progression and clinical outcomes require validation in larger prospective studies.

Microbiota-targeted interventions, including probiotics, prebiotics, and other microecological approaches, may represent potential adjunctive strategies in future PCAS management. Further research incorporating longitudinal clinical cohorts, integrated multi-omics analyses, and mechanistic investigations will be important to clarify host–microbiota interactions and evaluate the safety and efficacy of microbiota-based interventions during different stages of post-cardiac arrest care.

A better understanding of gut microbiota alterations following cardiac arrest may contribute to improved risk assessment and support development of novel adjunctive therapeutic approaches for patients with PCAS.
